# Climatic factors control the geospatial distribution of active ingredients in *Salvia miltiorrhiza* Bunge in China

**DOI:** 10.1038/s41598-018-36729-x

**Published:** 2019-01-29

**Authors:** Chenlu Zhang, Dongfeng Yang, Zongsuo Liang, Jingling Liu, Kaijing Yan, Yonghong Zhu, Shushen Yang

**Affiliations:** 1 College of Life Sciences, Northwest Agriculture & Forestry University, Yangling, 712100 P. R. China; 2 College of Biological Sciences & Engineering, Shaanxi University of Technology, Hanzhong, 723001 P. R. China; 3 College of Life Sciences, Zhejiang Sci-Tech University, Hangzhou, 310018 P. R. China; 4 Tasly Holding Group Co., Ltd, Tianjin, 300410 P. R. China

## Abstract

Climate change profoundly influences the geospatial distribution of secondary metabolites and causes the geographical migration of plants. We planted seedlings of the same species in eighteen ecological regions along a latitudinal gradient in eastern and western China, in order to explore the regulation of multi-climatic factors on active ingredient accumulation in *Salvia miltiorrhiza* Bunge. The correlations between six active ingredient contents and ten climatic factors were investigated to clarify their relationships. We found that climatic factors not only regulated active ingredient contents but also markedly influenced their composition and led to a specific geospatial distribution of these active ingredients in China. The main climatic factors include the air temperature, precipitation, atmospheric vapour pressure and sunshine duration. Future warming in high-latitude regions could cause continued northward expansion of planting areas suitable for *S. miltiorrhiza*. The effect of extreme climatic conditions on active ingredients should not be overlooked. The findings of this study can help farmers scientifically choose suitable cultivation regions in the future. Furthermore, this study provides an innovative idea for the exploration of secondary metabolic responses to changing ecological factors in medicinal plants.

## Introduction

Climate change profoundly influences the geospatial distributions of organisms and theirhabitats^1–3^. According to the “China blue book on climate change in 2018” issued by the China Meteorological Administration, from 1951 to 2017, the annual average and surface temperature increased by 0.24 °C every ten years; the rate of warming in the north was considerably higher than that in the south; the rate of warming in the west was higher than that in the east; extreme precipitation increased; extreme low temperature markedly decreased; and extreme high temperature notablyincreased^4^. Climate change has caused the geographical migration of Chinese medicinal plants^5,6^. *Salvia miltiorrhiza* Bunge (known as Danshen in China) belongs to the *Lamiaceae* family. For thousands of years, the roots of this species have been broadly used as an herbal medicine to treat cardiovascular and cerebrovascular diseases in China^7–9^. Currently, *S. miltiorrhiza* is more widely distributed (more than 18 provinces in China) than other internationally renowned traditional Chinese herbal medicines (TCHMs), such as *Panax ginseng* and *Panax pseudoginseng* var. *notoginseng*^5^. What has changed and how it has changed were less concerned in geographical migration of planting areas.

The main active ingredients in *S. miltiorrhiza* roots include water-soluble phenolic acids, e.g., rosmarinic acid (RA) and salvianolic acid (SAB) and fat-soluble diterpenoid quinines, e.g., dihydrotanshinone (DTS), cryptotanshinone (CTS), tanshinone I (TS I), and tanshinone IIA (TS IIA). These groups of compounds differ in clinical efficacy; thus, their proportions also affect their medicinal efficacy^7–11^. One study reported that phenolic acids and tanshinones can be used as quality markers to identify genotypes and locations and assess the quality of *S. miltiorrhiza*^12^. *S. miltiorrhiza* has been indexed in the United States pharmacopoeia, European pharmacopoeia, British pharmacopoeia, Japanese pharmacopoeia, and so on. Danshen capsules are an officially approved drug in the Netherlands. Compound Danshen dripping pills are currently being utilized as a drug in over twenty nations^13^. Over 90% of the market demands in China are supplied by cultivated products.

The active ingredients of medicinal plants with the same genotype are mainly determined by ecological conditions^12,14^. Many studies have shown that light, temperature, water, soil, and other ecological factors considerably influence the anabolism and accumulation of secondary metabolites^15–17^. Specific ecological environments govern the active ingredient compositions of medicinal plants.

The geo-herbalism region of Chinese herbal medicine has a strong relationship with ecological environments. A recent study reported that climatic factors contributed more to the active ingredient compositions in *Sinopodophyllum hexandrum* (Royle) T.S. Ying than did soil factors^18^. However, the influence of climate change on the geospatial distribution characteristics of active ingredients in *S. miltiorrhiza* remains unclear. In addition, the main climatic factor regulating active ingredient accumulation in *S. miltiorrhiza* and the relationships between these ingredients and different climate conditions have yet to be systemically studied.

Previous studies focused on variation in the active ingredients in *S.miltiorrhiza* collected from different regions or of different genotypes, changes at a limited spatial scale, and the variation in key genes and enzymes influenced by certain ecological factors^1,19–25^. Comprehensive analysis of active ingredient responses to ecological factors is complicated because of different genetic backgrounds, limited space, or ecological factor constraints. The geospatial distribution characteristics of active ingredients refer to the regular patterns of the active ingredients in some space^26^. In this study, we chose eighteen experimental sites along a latitudinal gradient in eastern and western China. The geographical range covered nearly all locations recorded in previous studies and our survey work. We analysed the changes in six active ingredients among geospatial locations and illustrated the geospatial distribution characteristics of active ingredients in *S. miltiorrhiza*. Correlation and multiple linear regression analyses revealed the key climatic factors controlling this geospatial distribution. Our conclusion provides scientific suggestions for the selection of suitable production areas under future climate change. The experimental design and research presented here provide novel ways to explore secondary metabolic responses to changes in ecological factors in medicinal plants.

## Material and Methods

### Site descriptions and plant materials

**Figure 1:**
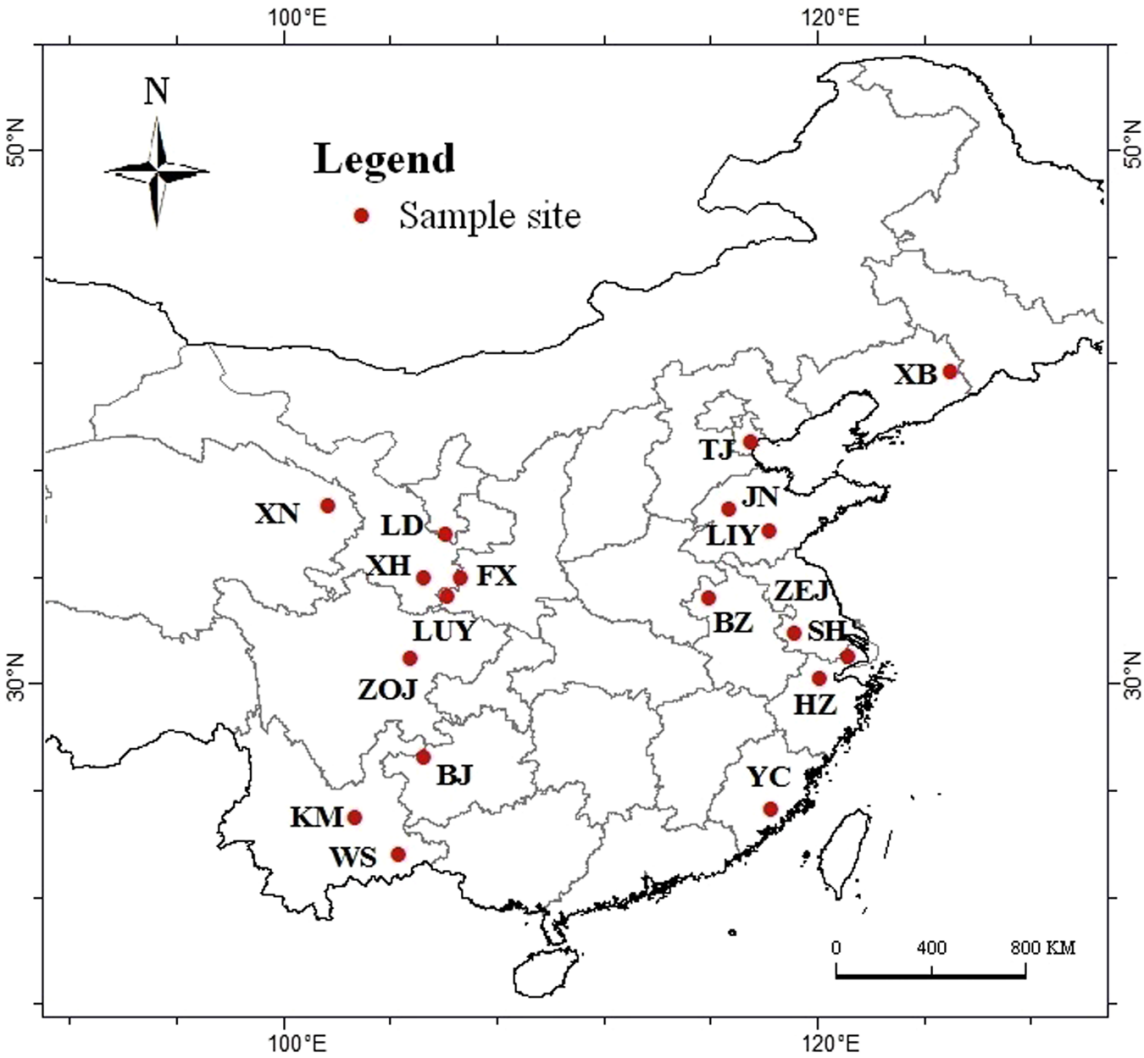
Growth locations of *S. miltiorrhiza* populations in China sampled for this study. Maps were generated using ArcGIS 10.0 (ESRI Inc. 2014).

The planting locations of *S. miltiorrhiza* in China were investigated from 2011 to 2012 by our team. According to the existing distribution, we transplanted seedlings of the same species into eighteen testing sites from April 10 to 30 in 2012. The experiment was repeated in 2013. We designed two vertically parallel lines of experimental sites along a latitudinal gradient. All locations are shown in Fig. 1 and described in Table 1. Every site, which included unshaded plots with moderate fertility soils and that received no extra fertilizer or water inputs, was managed based on uniform standards. Uniform seedlings were transplanted in an approximately 1000 square metre region at every site. We collected 50 individual samples with the scientific method at each site, divided them into five test samples on average (n = 10 within two year), and removed the rhizome. Fresh root samples were rapidly cleaned with flowing water, dried in a ventilated area, heated in a stove at a constant temperature of 50 °C, and then crushed into a fine powder that could pass through 65 mesh.

**Table 1:** Geographic position of the 18 testing sites in China.

Sample No.	Different Zones	Locations	Abbreviation	Longitude(°E)	Latitude(°N)	Elevation(m)
1	Eastern sites	Xinbin county, Fushun City, Liaoning Province	XB	125.04	41.73	340
2		Binghai District, Tianjin City	TJ	117.59	39.08	5
3		Changqin District, Jinan City, Shandong Province	JN	116.75	36.55	46
4		Luozhuang District, Linyi City, Shandong Province	LIY	118.22	35.77	66
5		Lixin county, Bozhou City, Anhui Province	BZ	115.96	33.25	28
6		Jurong county, Zhengjiang City, Jiangsu Province	ZEJ	119.17	31.95	27
7		Songjiang District, Shanghai City	SH	121.20	31.07	15
8		Jianggan District, Hangzhou City, Zhejiang Province	HZ	120.12	30.25	10
9		Yongchun county, Quanzhou City, Fujian Province	YC	118.30	25.32	138
10	Western sites	Chengbei District, Xining City, Qinhai Province	XN	101.75	36.69	2,304
11		Longde county, Guyuan City, Ningxia Province	LD	106.12	35.62	2,090
12		Xihe county, Longnan City, Gansu Province	XH	105.30	34.01	1,587
13		Fengxian county, Baoji City, Shaanxi Province	FX	106.66	33.97	1,244
14		Lueyang county, Hanzhong City, Shaanxi Province	LUY	106.16	33.34	870
15		Zhongjiang county, Deyang City, Sichuan Province	ZOJ	104.77	30.97	468
16		Qixingguan District, Bijie City, Guizhou Province	BJ	105.31	27.30	1,482
17		Guandu District, Kunming City, Yunnan Province	KM	102.74	25.02	2,238
18		Yanshan county, Wenshan City, Yunnan Province	WS	104.35	23.61	1,542

Voucher specimens from all populations were identified by Professor Yuejin Zhang of Northwest A&F University through comparison with herbarium specimens of *S. miltiorrhiza* located at Northwest Agriculture & Forestry University (WUK381982-5143). The seedlings were obtained from the Good Agriculture Practice production base belonging to Shaanxi Tasly Plant Pharmaceutical Co. Ltd. (Shangluo City, Shaanxi Province, China), which has been authenticated three times by China’s Food and Drug Administration.

### Related climatic factors

Data on annual climatic factors were obtained from the China Meteorological Data Sharing Service System^27^ and Provincial Agricultural Meteorological Service Center. The 10 main climatic factors included annual extreme high temperature (AEHT), annual precipitation (AP), annual average barometric pressure (AABP), annual average wind speed (AAWS), annual average temperature (AAT), annual average vapour pressure (AAVP), annual average relative humidity (AARH), annual average lowest temperature (AALT), annual accumulated temperature above 10 °C (AAT ≥ 10), and annual sunshine duration (ASD) from 2012 to 2013 (Table 2). Climate factors were averaged over two years.

**Table 2:** Climatic factors examined in this study.

Sample No.	Abbreviation	AEHT(°C)	AP(mm)	AABP(hpa)	AAWS(m·s^−1^)	AAT(°C)	AAVP (hpa)	AARH (%)	AALT (°C)	ACT ≥ 10 (°C)	ASD(h)
1	XB	33.5	756	1,002	2.2	6.7	10.0	71.4	1.0	2,850	2,334
2	TJ	37.1	575	1,016	2.8	12.8	11.0	61.0	8.4	4,350	2,440
3	JN	38.2	736	996	2.7	14.7	11.7	62.0	10.7	4,488	2,408
4	LIY	36.8	871	1,004	2.4	13.1	12.6	67.4	8.5	4,276	2,245
5	BZ	38.6	726	1,012	2.9	16.2	13.0	61.9	11.9	4,690	2,337
6	ZEJ	40.1	1,098	1,012	2.9	16.8	14.9	68.1	12.9	5,002	2,197
7	SH	39.2	1,380	1,016	1.1	18.0	16.9	72.3	15.2	5,283	1,532
8	HZ	38.5	1,729	1,011	2.1	17.1	15.5	70.8	14.0	5,230	1,521
9	YC	39.2	1,877	990	3.3	20.9	18.1	71.2	17.0	7,215	1,814
10	XN	32.6	412	770	1.0	6.1	6.1	55.2	−0.7	2,855	2,661
11	LD	29.8	706	824	2.2	5.1	7.3	54.9	3.5	2,170	2,623
12	XH	33.7	538	842	2.5	8.8	10.2	73.0	4.7	3,426	1,842
13	FX	37.6	777	905	1.1	12.1	12.0	68.0	7.1	3,715	1,923
14	LUY	35	805	924	1.6	13.5	13.1	75.3	9.8	4,244	1,374
15	ZOJ	37.5	898	963	1.5	16.5	14.6	79.2	13.9	5,010	996
16	BJ	33.6	783	848	1.1	14.2	12.7	75.1	10.8	3,840	1,339
17	KM	30.9	862	809	2.8	15.1	12.6	70.8	11.5	5,320	2,354
18	WS	31.8	923	844	3.5	16.4	14.4	77.6	12.8	5,780	2,050

Data were obtained from the China Meteorological Data Sharing Service System and Provincial Agricultural Meteorological Service Center. Abbreviations: AEHT-annual extreme high temperature, AP-annual precipitation, AABP-annual average barometric pressure, AAWS-annual average wind speed, AAT-annual average temperature, AAVP-annual average atmospheric vapour pressure, AARH-annual average atmospheric relative humidity, AALT-annual average lowest temperature, ACT ≥ 10-annual cumulative temperature above 10 °C, ASD-annual sunshine duration.

### Chromatographic detection analysis by reverse-phase high-performance liquid chromatography (RP-HPLC)

RA, SAB, DTS, CTS, TSI, and TSIIA were determined by HPLC as described by Peng L *et al*.^25^. The sum of RA and SAB defined the total phenolic acid (TPA), and the sum of CTS, TSI, and TSIIA defined the total tanshinone (TTS). The effectiveness, linearity, repeatability, precision, and stability of the HPLC method were confirmed to be sufficient. The results of chromatographic analysis were obtained from the Institute of Pharmacy Engineering (Northwest Agriculture & Forestry University, Yangling, China).

### Statistical analysis

The results are presented as the mean ± standard deviation (SD). The data were analysed by one-way ANOVA and Duncan’s multiple comparison test using IBM SPSS Statistics 23 software. Statistical significance was considered as P < 0.05. Pearson’s two-tailed correlation analysis (CA), principal component analysis (PCA), and stepwise multiple linear regression (SMLR) analysis were performed using IBM SPSS Statistics 23 software^18^. The complex relationships between ecological factors and active ingredient contents were intuitively displayed by a heat map using HemI 1.0 software^28^.

## Results

### Geospatial distribution characteristics of tanshinones

**Figure 2:**
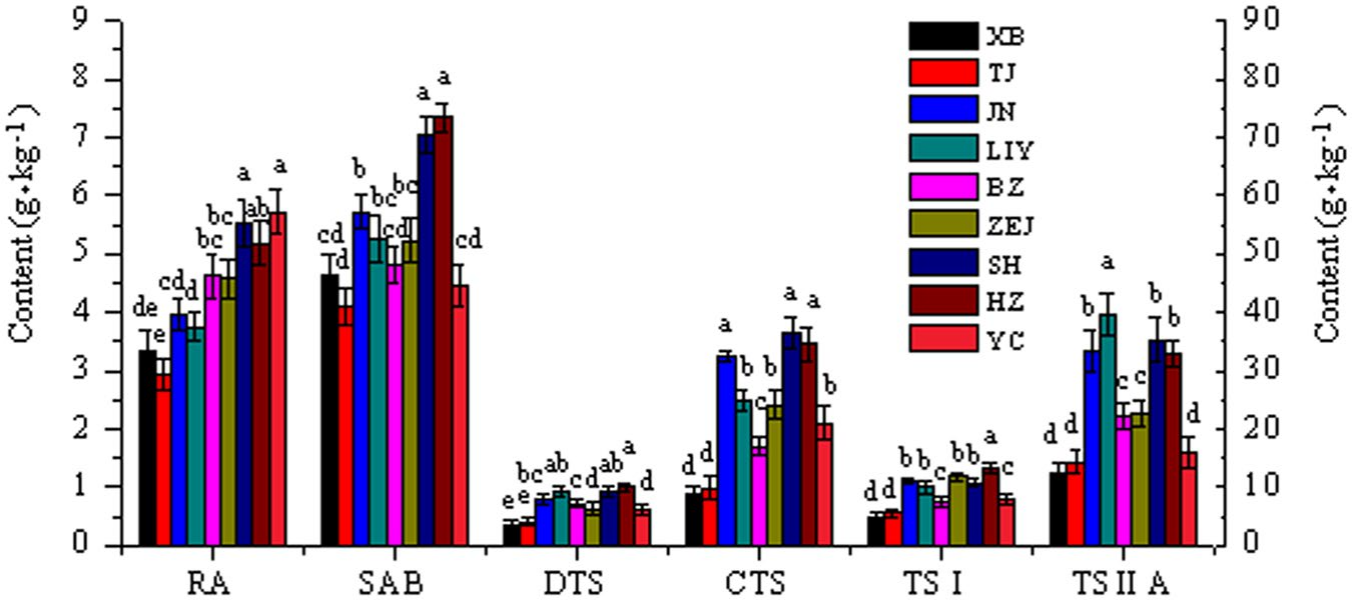
Active ingredient contents of *S. miltiorrhiza* in the eastern sites. The SAB content is presented on the right Y axis, and the other ingredient contents are presented on the left Y axis. For each location, values with different letters are significantly different according to Duncan’s test (P < 0.05), n = 10, mean ± SD. This figure was created using OriginPro 8 software. RA-rosmarinic acid, SAB-salvianolic acid B, DTS-dihydrotanshinone, CTS-cryptotanshinone, TSI-tanshinone I, TSIIA-tanshinone IIA.

**Figure 3:**
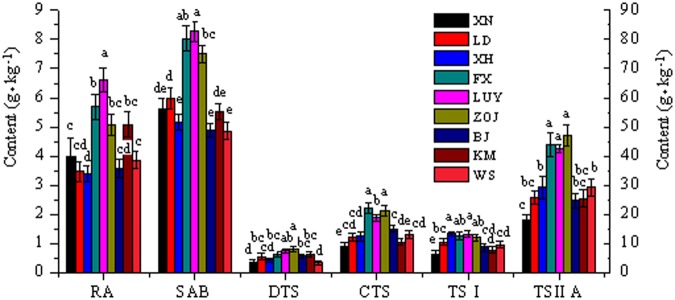
Active ingredient contents of *S. miltiorrhiza* in the western sites. The SAB content is presented on the right Y axis, and the other ingredient contents are presented on the left Y axis. For each location, values with different letters are significantly different according to Duncan’s test (P < 0.05), n = 10, mean ± SD. This figure was created using OriginPro 8 software. RA-rosmarinic acid, SAB-salvianolic acid B, DTS-dihydrotanshinone, CTS-cryptotanshinone, TSI-tanshinone I, TSIIA-tanshinone IIA.

**Figure 4:**
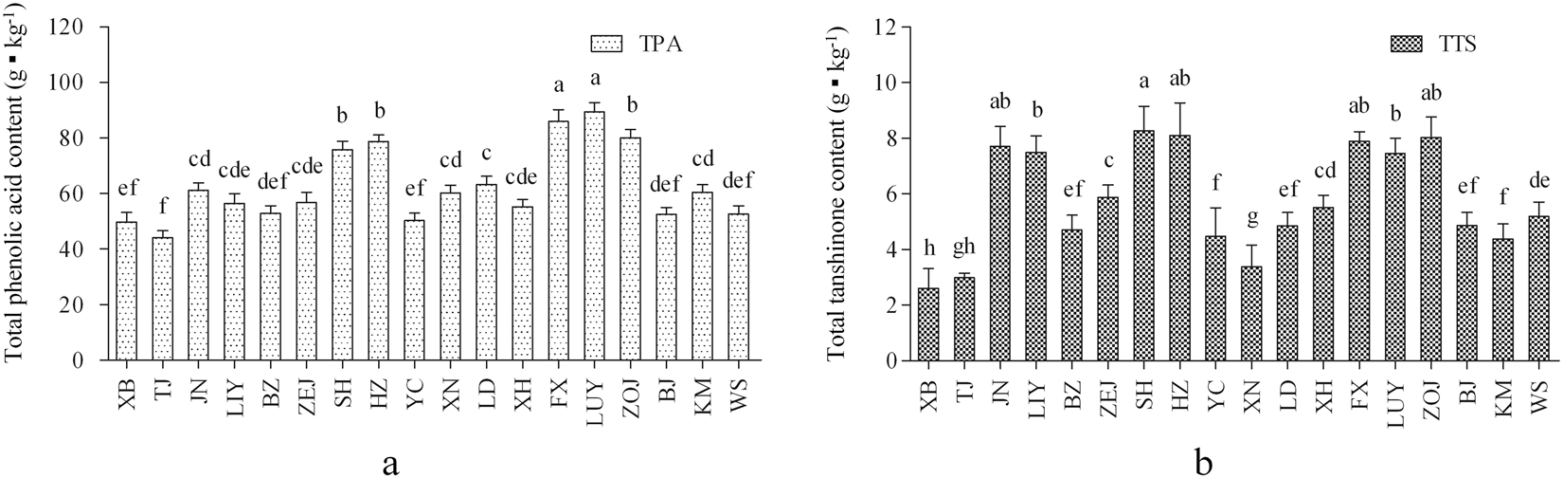
Total phenolic acid and total tanshinone contents in *S. miltiorrhiza* roots. (**a**) Results from the eastern sites; (**b**) results from the western sites. For each site, values followed by different letters are significantly different according to Duncan’s test (P < 0.05), n = 10, mean ± SD. This figure was created using GraphPad Prism software. TTS-total tanshinone, TPA-total phenolic acid.

The active ingredient contents of *S. miltiorrhiza* grown in the 18 test sites are shown in Figs 2–4. The results showed significant differences between the eastern and western sites. The change in single-component contents was similar to that in the total same type component contents. With a decrease in latitude, the TTS content showed a double peak in the eastern sites but a single peak in the western sites. Although the variation in TTS content was different between the eastern and western sites, the average TTS content was similar between these sites. The TTS contents in the western high-altitude and mid-latitude sites were evidently higher than those in the eastern parallel sites.

### Geospatial distribution characteristics of phenolic acids

The variation in TPA content was similar to that in TTS content in different test sites (Figs 2–4). In the eastern sites, the SAB content showed a double peak with decreasing latitude. The SAB and RA contents presented single-peak changes in the western sites. The TPA content gradually increased with decreasing latitude in the eastern sites, except for YC. The TPA content was high in the western mid-latitude sites. The average TPA content in the western sites was significantly higher than that in the eastern sites. The TPA contents in the western high-altitude and mid-latitude sites were evidently higher than those in the parallel eastern sites.

### Geospatial distribution characteristics of the proportion of active ingredients

**Figure 5:**
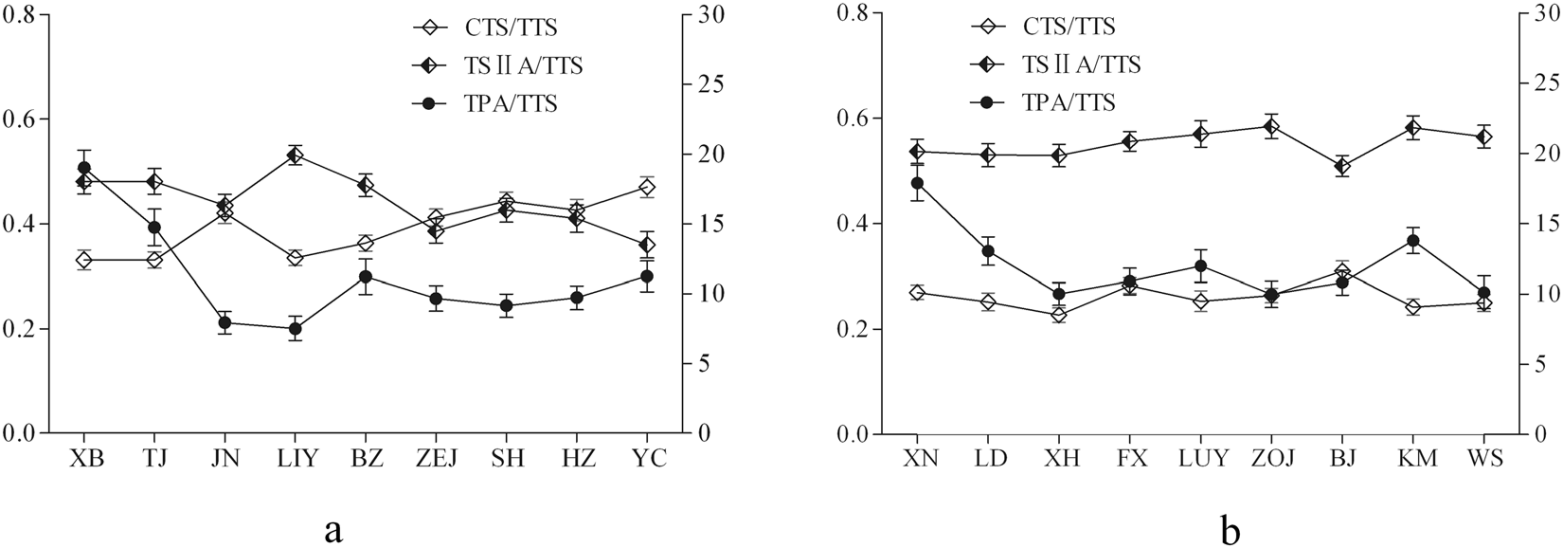
Composition proportion of tanshinones in *S. miltiorrhiza* roots. (**a**) Eastern sites; (**b**) western sites. The CTS/TTS and TSIIA/TTS content proportions are presented on the left Y axis, and the TPA/TTS content proportion is presented on the right Y axis. n = 10, mean ± SD. This figure was created using GraphPad Prism software. CTS-cryptotanshinone, TSIIA-tanshinone IIA, TTS-total tanshinone, TPA-total phenolic acid.

The active ingredients in *S. miltiorrhiza* mainly include water-soluble phenolic acids and fat-soluble tanshinones. Many studies have shown that phenolic acids and tanshinones are synthesized through different biosynthetic pathways in *S. miltiorrhiza*^29,30^. The proportion of active ingredients affects pharmacological activities^8,9^. This study focused on the changes in the CTS/TTS, TSIIA/TTS, and TPA/TTS proportions among different geospatial areas (Fig. 5). With decreasing latitude, the curves of the CTS/TTS and TSIIA/TTS proportions exhibited cross-fluctuation trends in the eastern sites, and the curves of the CTS/TTS proportion presented an increasing trend in the eastern sites. However, the CTS/TTS proportion was always significantly lower than the TSIIA/TTS proportion in the western sites. The proportion of tanshinones changed less in western mid-latitude sites. Interestingly, the CTS/TTS proportion in the western sites was always lower than that in the eastern sites, but the TSIIA/TTS proportion in the western sites was higher than that in the eastern sites. Therefore, targeted planting areas could be selected according to these relationships.

The TPA/TTS value ranged from 7.52 to 19.02 in the eastern sites and from 9.97 to 17.88 in the western sites (Fig. 5). In the eastern and western sites, the trend of the TPA/TTS proportion resembled an inverted letter L. This result indicated that the TPA/TTS proportion decreased with latitude and rapidly declined in the high-latitude sites but slowly changed in the mid-and low-latitude sites. This result may be due to the high TPA or low TTS content in the high-latitude areas.

### PCA of the active ingredients

**Figure 6:**
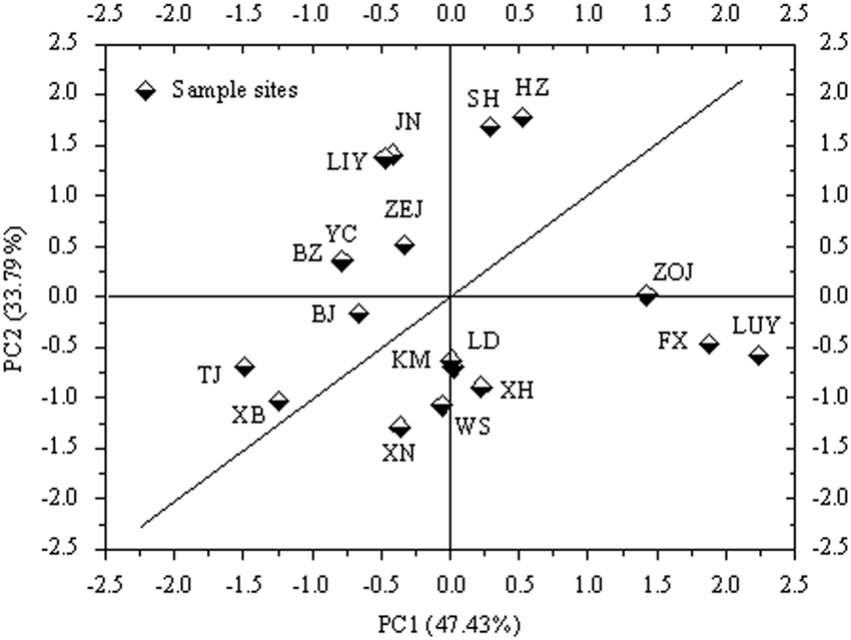
PCA scatter plot of the active ingredient contents. The horizontal axis depicts PC1, mainly representing phenolic acids, TSI and TSIIA content, and accounts for 47.43% of the variation. The vertical axis depicts PC2, mainly representing DTS and CTS content, and accounts for 33.79% of the variation. This figure was created using OriginPro 8 software.

PCA is a common multivariate statistical method adopted to investigate correlations between multiple variables. PCA can make use of a few principal components to reveal the internal structure of multiple variables^25^. In the PCA of the six compounds, the first two principal components were selected. The contribution of the eigenvalues of these two components reached 81.22% based on a rotated component matrix with varimax Kaiser normalization. A 2D PCA scatter plot was used to visualise the geospatial distribution characteristics of the active ingredient contents. PC1 mainly represented the RA, SAB, TSI, and TSIIA contents, which accounted for 47.43% of the variation. PC2 mainly represented the DTS and CTS contents, which accounted for 33.79% of the variation (Fig. 6 and Table 3). Furthermore, similar characteristics were found for the active ingredient contents and proportions. Excluding TJ and XB, six other eastern sites had higher DTS and CTS contents than all western sites. The lowest active ingredient content was found in TJ and XB. The high active ingredient content was found in ZOJ, FX and LUY, which are located in the western mid-latitude regions. The sites above the diagonal line in Fig. 6 had higher DTS and CTS contents than those under the diagonal line.

**Table 3:** Rotated component matrix of the principal component analysis.

Active ingredients	PC1	PC2
RA	0.700	0.283
SAB	0.928	0.216
DTS	0.364	0.887
CTS	0.287	0.925
TSI	0.761	0.379
TSIIA	0.837	0.339

RA-rosmarinic acid content, SAB-salvianolic acid B content, DTS-dihydrotanshinone content, CTS-cryptotanshinone content, TSI-tanshinone I content, TSIIA-tanshinone IIA content.

### Correlation analysis between active ingredient contents and climatic factors

**Figure 7:**
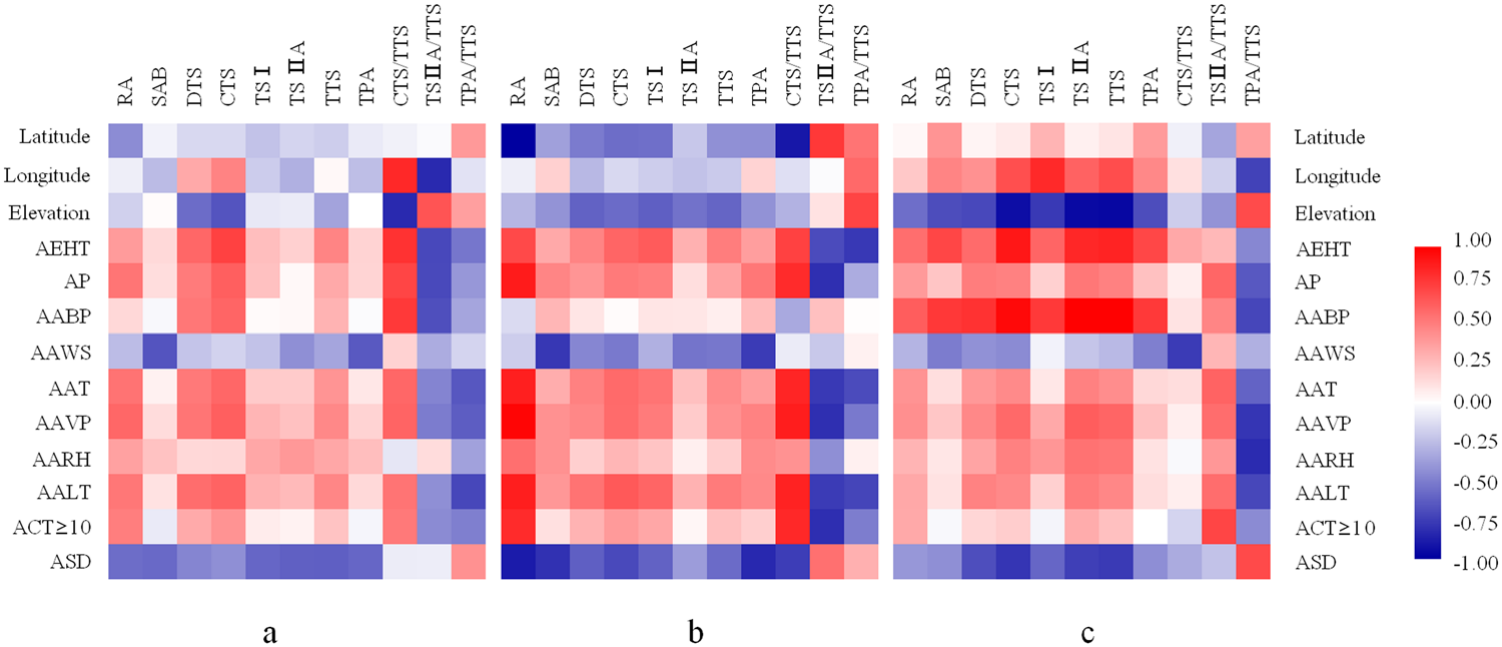
Correlations between the ecological factors and active ingredient contents of *S. miltiorrhiza*. (**a**) Eastern and western sites, (**b**) Eastern sites, and (**c**) Western sites. Pearson’s two-tailed correlation analysis was performed using IBM SPSS Statistics 23 software. The heat map displaying the correlation coefficients between ecological factors and active ingredient contents was created using HemI 1.0 software. AEHT-annual extreme high temperature, AP-annual precipitation, AABP-annual average barometric pressure, AAWS-annual average wind speed, AAT-annual average temperature, AAVP-annual average atmospheric vapour pressure, AARH-annual average atmospheric relative humidity, AALT-annual average lowest temperature, ACT ≥ 10-annual cumulative temperature above 10 °C, ASD-annual sunshine duration. RA-rosmarinic acid, SAB-salvianolic acid B, DTS-dihydrotanshinone, CTS-cryptotanshinone, TSI-tanshinone I, TSIIA-tanshinone IIA, TTS-total tanshinone, TPA-total phenolic acid.

Results from the correlation analysis between the active ingredient contents in *S. miltiorrhiza* and ecological factors are shown in Fig. 7. In the eastern and western sites, the active ingredient contents were positively correlated with the AALT, AAT, AEHT, AP, and AAVP (Fig. 7a, Table S1). The active ingredient contents showed a significant negative correlation with ASD, especially tanshinones in the western sites (P < 0.05) and phenolic acids in the eastern sites (P < 0.05) (Table S2, Table S3). In the western sites, the tanshinone content showed a significant negative correlation with elevation and a significant positive correlation with longitude (Fig. 7c, Table S3). The results showed that appropriate air temperature and moisture conditions were conducive to the accumulation of phenolic acids and tanshinones in *S. miltiorrhiza*. Excessive sunshine did not improve the accumulation of these ingredients.

### Correlation analysis between active ingredient proportion and climatic factors

Results from the correlation analysis between the active ingredient proportion and climatic factors are shown in Fig. 7. The CTS/TTS proportion showed a significant positive correlation with the AEHT, AP, AAT, AAVP, AALT, and ACT ≥ 10 (P < 0.01) in the eastern sites, whereas the TSIIA/TTS proportion was negatively correlated with these factors (P < 0.01) (Fig. 7b, Table S2). The CTS/TTS proportion was less influenced by these factors, but the TSIIA/TTS proportion exhibited a significant positive correlation with these factors in the western sites (Fig. 7c, Table S3). These results indicated that the accumulation of tanshinones increased under relatively warm and moist climatic conditions. High temperature and excessiverain would observably reduce the TSIIA/TTS proportion but increase the CTS/TTS proportion.

Interestingly, ASD was negatively correlated with the active component contents. ASD was also negatively correlated with the CTS/TTS proportion (P < 0.05) but positively correlated with the TSIIA/TTS proportion in the eastern sites (Fig. 7b, Table S2). However, ASD exhibited a weak negative correlation with the CTS/TTS or TSIIA/TTS proportion in the western sites (Fig. 7c, Table S3). This finding indicated that a high ASD increased the TSIIA/TTS proportion in eastern high-latitude coastal areas but decreased it in western high-latitude areas because of an extremely long ASD. At the maximum spatial scale, the CTS/TTS proportion showed a significant negative correlation with elevation (P < 0.01) and a significant positive correlation with longitude (P < 0.05). However, the correlation of the TSIIA/TTS proportion with elevation or longitude was exactly the opposite (Fig. 7a, Table S1).

In addition, the TPA/TTS proportion generally increased with increasing ASD in the eastern and western sites, especially in the western sites. The TPA/TTS proportion decreased with an increase in air temperature and the AAVP. The TPA/TTS proportion showed a significant negative correlation with the AEHT and AALT (P < 0.05) in the eastern sites and with the AAVP, AARH, and AABP (P < 0.05) in the western sites. The TPA/TTS proportion increased with latitude and elevation (Fig. 7). These results indicated that the TPA/TTS proportion would increase under low-temperature stress, water stress, or an excessive ASD.

### Stepwise multiple linear regression (SMLR) analysis

SMLR has been applied to identify the best prediction model in many studies. The standardized regression coefficient reveals the significance of an individual descriptor in the regression model. A high absolute value of this coefficient corresponds to a high weight of variables in the model^31,32^. We used SMLR analysis to establish two prediction models of the tanshinone proportion (Table 4). The CTS/TTS proportion showed a positive linear correlation with the AEHT, AP, and ASD, but the TSIIA/TTS proportion exhibited a negative linear correlation with these factors. This finding demonstrated that extreme climatic conditions, such as excessive high temperature, excessive rainfall, and excessive sunshine, could cause the CTS/TTS proportion to increase while causing the TSIIA/TTS proportion to decrease. The absolute values of the standardized regression coefficients ranked as follows: AEHT > AP > ASD (for CTS/TTS) and AP > AEHT > ASD (for TSIIA/TTS). Therefore, the proportion of tanshinones can be considered to be mainly determined by the AEHT, AP, and ASD. The AEHT had the strongest positive influence on the CTS/TTS proportion, followed by AP and ASD. AP had the strongest negative effects on the TSIIA/TTS proportion, followed by the AEHT. The influence of ASD on the CTS/TTS and TSIIA/TTS proportions was weaker than the influence of AP and AEHT.

**Table 4:** Stepwise multiple linear regression (SMLR) analysis of climatic factors influencing the active ingredients in *S. miltiorrhiza* roots.

No.	Stepwise multiple linear regression model	*R* ^2^	*F*	*P*
Model I	*Z*_*CTS/TTS*_ = 0.611*Z*_*AEHT*_ + 0.547*Z*_*AP*_ + 0.364*Z*_*ASD*_	0.845	25.448	0.000
Model II	*Z*_*TSIIA/TTS*_ = −0.496*Z*_*AEHT*_ − 0.591*Z*_*AP*_ − 0.448*Z*_*ASD*_	0.738	13.113	0.000

The value of Zx is a standardized dimensionless number. AEHT-annual extreme high temperature, AP-annual precipitation, ASD-annual sunshine duration. SMLR analysis was performed using IBM SPSS Statistics 23 software. Collinearity statistics: VIF ≤ 1.5.

## Discussion

Most plants can alter their metabolism to adapt to environmental changes. The characteristics of plant secondary metabolites in certain ecological zones can reflect the characteristics of the ecological environment^33–35^. Climate change profoundly influences the geospatial distribution of secondary metabolites and causes the geographical migration of Chinese medicinal plants^5,6,26^. This study provided sufficient evidence to discover the geospatial distribution characteristics of active ingredients and illuminated the regulatory effects of key climatic factors on the active ingredients of *S. miltiorrhiza* in a large geographical range in China.

### Active ingredients of *S. miltiorrhiza* have evident geospatial distribution characteristics in China

In our study, the evident geospatial distribution characteristics of active ingredients in *S. miltiorrhiza* were found in China. High TTS and TPA contents were observed in the sites between eastern mid- and high-latitude, the sites between eastern low- and mid-latitude, and western mid-latitude sites. The TTS and TPA contents in the western high-altitude and mid-latitude sites were significantly higher than those in the eastern sites with a corresponding latitude. The TPA/TTS proportion decreased with decreasing latitude and rapidly declined in the high-latitude sites. The TSIIA/TTS proportion was highest in the eastern mid- to high-latitude sites, but the CTS/TTS proportion was adversely affected there. The CTS/TTS proportion was always significantly lower than the TSIIA/TTS proportion in the western sites. The CTS/TTS proportion in the western sites maintained a lower level than that in the eastern sites at the similar latitude locations, but the distribution of the TSIIA/TTS proportion showed the opposite pattern (Fig. 1, Fig. 5).

The PCA results also showed that the DTS and CTS contents in the eastern sites were generally higher than those in the western sites, except for TJ and XB. High RA, SAB, TSI, and TSIIA contents were observed in ZOJ, FX, and LUY (Fig. 6). Therefore, planting areas can be targeted based on the geospatial distribution characteristics of active ingredients in *S. miltiorrhiza*. The composition of active ingredients was strongly related to the climatic conditions, which can also reflect the characteristics of the ecological environment.

### Climate-induced stress changes the proportion of active ingredients in *S. miltiorrhiza*

Through analysis of our experimental results, we found that climatic conditions strongly influenced the proportion of active ingredients, especially extreme temperature, excessive rainfall, and excessive sunshine. Extreme temperature was the most important factor affecting the proportion of active ingredients in *S. miltiorrhiza*. SMLR models also showed that the AEHT was linearly and negatively correlated with the TSIIA/TTS proportion but linearly and positively correlated with the CTS/TTS proportion (Table 4). Correlation analysis showed that AP exhibited a significant negative correlation with the TSIIA/TTS proportion and a positive correlation with the CTS/TTS proportion in the eastern sites (Fig. 7b, Table S2). However, the proportion of tanshinones was slightly influenced by AP in the western sites (Fig. 7c, Table S3). These results demonstrated that eastern extreme precipitation can reduce the TSIIA/TTS proportion while simultaneously increasing the CTS/TTS proportion. Therefore, we hypothesize that these key factors may have caused the transformation between TSIIA and CTS.

Sunshine plays an important role in plant growth and the synthesis of active ingredients in medicinal plants^36–38^. Previous studies reported that *S. miltiorrhiza* has a strong adaptability to low-light conditions and exhibits photoinhibition in response to excessive light^38^. These physiological characteristics are crucial for adaptation to different ecological environments in *S. miltiorrhiza*. In the present study, the active ingredient content decreased with increasing ASD in both the eastern and western sites. The ASD range was from 1521 h to 2440 h in the eastern sites and from 996 h to 2661 h in the western sites, as shown in Table 2. A longer ASD did not increase the TSIIA/TTS and CTS/TTS proportions in the western high-altitude regions, possibly due to the excessive ASD, which inhibited the biosynthesis and accumulation of tanshinones there. SMLR models revealed that an excessive ASD evidently reduced the TSIIA/TTS proportion and increased the CTS/TTS proportion. The influence of ASD was weaker than that of the AEHT and AP (Table 4). The TPA/TTS proportion decreased with increasing temperature and AAVP. The increase in ASD increased the TPA/TTS proportion in the eastern and western sites, especially in the western sites. The AEHT and AALT were the key factors influencing the TPA/TTS proportion in the eastern sites (Fig. 7). Furthermore, water was the main factor influencing the TPA/TTS proportion in the western sites, and drought stress significantly increased this effect. Therefore, we also found that the TPA/TTS proportion increased with increasing latitude and elevation.

### Temperature was the primary climatic factor influencing tanshinone content and proportion

Temperature is crucial in plant secondary metabolism^14,33^. Our previous study also found that the annual average 0–20 cm ground temperature and average temperature showed a significant positive correlation with the TTS content in 0.3–0.6 cm diameter roots of *S. miltiorrhiza* in Shaanxi Province. The tanshinone content and proportion were influenced by temperature^39^. An increase in temperature promoted the accumulation of tanshinones in this study. In addition, SMLR models revealed that the AEHT was the primary factor influencing the proportion of tanshinones (Table 4). This result indicated that the ability of TSIIA to tolerate temperature stress is inferior to that of CTS. The accumulation of TSIIA probably requires a narrower and more adaptable temperature range than the accumulation of CTS. In conclusion, temperature is the primary climatic factor affecting the accumulation of tanshinones.

### Water was the key ecological factor influencing phenolic acid content

The average TPA content in the western sites was approximately 1.14 times higher than that in the eastern sites (Fig. 4), and the average AP in the western sites was 745 mm, i.e., only up to 68.77% of that in the eastern sites (Table 2). Moreover, low water conditions could increase the accumulation of phenolic acids in *S. miltiorrhiza*. This result was consistent with those of Liu HY *et al*. and Liu DH *et al*.^15,40^. The TPA content was negatively correlated with latitude in the eastern sites but positively correlated with latitude in the western sites. The maximum difference in annual precipitation was 1302 mm among the eastern sites and 511 mm among the western sites (Table 2). In similar latitude sites in northern China, the phenolic acid content in the eastern sites was significantly lower than that in the western sites. This finding indicated that relatively wet and cold climatopes stimulated the accumulation of phenolic acids less than did relatively cold and arid climatopes. Many studies have also revealed that water regulates the accumulation of active ingredients in *S. miltiorrhiza*. The five other active constituent contents increased under intermediate water-stress conditions. Water stress significantly increases the yield of SAB and decreases that of TSIIA^15,39–41^. Therefore, scarce or excessive water could significantly stimulate the accumulation of phenolic acids in *S. miltiorrhiza* roots. Water is the most important climatic factor regulating the accumulation of phenolic acids in *S. miltiorrhiza*.

### Adverse climate may change the transformation of CTS and TSIIA

It has been reported that the structure of CTS and TSIIA only differs in the C_15_-C_16_ of the furan ring, which is a single or double bond. It has been demonstrated that CTS and TSIIA can transform mutually through catalytic hydrogenation^42–44^. The pharmacological actions of CTS and TSIIA are reportedly different. Analysis of the structure-effect relationship showed that the single bond of CTS increases antibacterial activity compared to the double bond of TSIIA^44^. We surmise that this phenomenon is related to plant stress resistance. An incompatible relationship between the TSIIA/TTS and CTS/TTS proportions was found in the present study. The SMLR results also showed that the CTS/TTS proportion exhibited a positive linear correlation with the AEHT, AP, and ASD, but the TSIIA/TTS proportion exhibited a negative linear correlation with these factors (Table 4). Therefore, the influence of climatic factors on component proportions may be associated with the key biotransformation reaction. Therefore, climatic stress may influence the biotransformation of CTS and TSIIA in *S. miltiorrhiza*, for example, excessive temperature, excessive rainfall or excessive sunshine duration.

### Climate change influences the geo-authenticity and targeted cultivation of *S. miltiorrhiza*

The China Meteorological Administration announced that extreme weather has increased, with the annual average land surface temperatures increasing by 0.24 °C every ten years and the warming rate increasing in western and northern China for nearly 60 years^4^. With the dynamic change in climate, the geo-authentic area of traditional Chinese medicinal materials has continued to migrate from its historical location^5,6,45^. The geo-authenticity of medicinal plant is the result of medicinal plants adapting to ecological environments. This concept has important ecological significance. Geo-authenticity is also used as a quality evaluation index for medicinal materials^5,46^. Latitude, longitude, and elevation change through space and govern the ecological environment^32,46^. At the maximum spatial scale, the TTS content was negatively correlated with elevation, and the TPA content was negatively correlated with longitude. The CTS/TTS and TSIIA/TTS proportions were strongly correlated with longitude and elevation, and the TPA/TTS proportion was negatively correlated with latitude and elevation.

Importantly, comprehensive assessment revealed a high active ingredient content in Chinese mid-latitude and low- to mid-latitude zones, such as Shandong Province, Henan Province, Shaanxi Province, Sichuan Province, Jiangsu Province, Yunnan Province, Guizhou Province, and so on. This finding was consistent with that of Chen SL *et al*.^46^. However, the warming of average land surface temperatures in high-latitude regions may cause northward expansion of geo-authentic areas in China^4,6,28,47^. Increasing extreme climatic events may strongly influence the active ingredient composition in *S. miltiorrhiza*. Our study revealed the geospatial distribution characteristics of active ingredient contents in *S. miltiorrhiza* and clarified their relationships with climate change. Therefore, scientific targeted cultivation of *S. miltiorrhiza* is feasible throughout China.

## Conclusion

In this study, the geospatial distribution characteristics of active ingredients in *S. miltiorrhiza* roots harvested from 18 different ecological regions in China were revealed. High TTS and TPA contents were observed in the sites between eastern mid- and high-latitude, the sites between eastern low- and mid-latitude, and western mid-latitude sites. The TTS and TPA contents in the western high-altitude and mid-latitude sites were significantly higher than those in the eastern sites of similar latitude. The CTS/TTS proportion in the western sites was lower than that in the eastern sites of similar latitude, but the change in the TSIIA/TTS proportion was reversed.

Adverse climatic conditions, especially extreme temperature, excessive rainfall, and excessive sunshine, could strongly influence the proportion of active ingredients. Extreme climatic conditions could reduce the TSIIA/TTS proportion while increasing the CTS/TTS proportion. The TPA/TTS proportion would decrease with increasing temperature and AAVP but would increase with increasing ASD. Suitable temperature, water, and sunshine would increase the accumulation of tanshinones. Scarce or excessive water can significantly stimulate the accumulation of phenolic acids but is not conducive to the accumulation of tanshinones in *S. miltiorrhiza*.

Temperature was the primary climatic factor affecting tanshinone content and proportion, while water strongly influenced the phenolic acid content. At the maximum spatial scale, the TTS content was negatively correlated with elevation, the TPA content was negatively correlated with longitude, the CTS/TTS and TSIIA/TTS proportions were strongly correlated with longitude and elevation, and the TPA/TTS proportion was negatively correlated with latitude and elevation.

In summary, climatic factors control the geospatial distribution characteristics of active ingredient contents in *S. miltiorrhiza* in China. The main climatic factors include the air temperature, precipitation, atmospheric vapour pressure and sunshine duration. Climate change is the most important reason for the migration of the *S. miltiorrhiza* cultivation region. Future warming in high-latitude regions may cause the planting regions suitable for *S. miltiorrhiza* to continue to expand northward. However, special attention should be paid to the influence of extreme climatic conditions on active ingredients. Targeted cultivation based on the geospatial distribution characteristics of active ingredients in *S. miltiorrhiza* should be taken seriously.

## Electronic supplementary material

Dataset 1
