# Supplementary material for: Climatic factors control the geospatial distribution of active ingredients in Salvia miltiorrhiza Bunge in China
Source: Sci Rep. 2019 Jan 29;9:904. doi: 10.1038/s41598-018-36729-x (PMC6351527; doi:10.1038/s41598-018-36729-x)
Supplement: Supplementary file 1 — Dataset 1 [file 41598_2018_36729_MOESM1_ESM.pdf]

Climatic factors control the geospatial distribution of active ingredients in *Salvia miltiorrhiza* Bunge in China

Authors

1st author Zhang Chenlu[a,b] chenluzhang_316@163.com

2nd author Yang Dongfeng[c] ydf807@163.com

3rd author *Liang ZongSuo[a,c] liangzs@ms.iswc.ac.cn

4th author Liu Jinglin[a] jinglingliu-sm@nwsuaf.edu.cn

5th author Yan Kaijing[d] kjyan@tasly.com.cn

6th author Zhu Yonghong[d] yhzhu@tasly.com.cn

7th author *Yang Shushen[a] yangshushen2014@163.com

*Correspondence to [liangzs@ms.iswc.ac.cn & yangshushen2014@163.com]

Author affiliations

a College of Life Sciences, Northwest Agriculture & Forestry University, Yangling 712100, P. R. China

b College of Biological Science & Engineering, Shaanxi University of Technology, Hanzhong 723001, P. R. China

c College of Life Sciences, Zhejiang Sci-Tech University, Hangzhou 310018, P. R. China

d Tasly Holding Group Co., Ltd., Tianjin 300410, P. R. China

Supplementary data

Table S1 Correlations between the ecological factors and active ingredient contents of *S. miltiorrhiza* cover the eastern and western sites

Table S2 Correlations between the ecological factors and active ingredient contents of *S. miltiorrhiza* in the eastern sites

Table S3 Correlations between the ecological factors and active ingredient contents of *S. miltiorrhiza* in the western sites

Eastern and western sites	RA	SAB	DTS	CTS	TS Ⅰ	TS ⅡA	TTS	TPA	CTS/TTS	TS ⅡA/TTS	TPA/TTS
Latitude	-0.410	-0.031	-0.126	-0.128	-0.207	-0.143	-0.166	-0.060	-0.032	0.000	0.397
Longitude	-0.042	-0.234	0.340	.478*	-0.169	-0.274	0.044	-0.224	.817**	-.776**	-0.090
Elevation	-0.153	0.033	-.527*	-.616**	-0.065	-0.054	-0.322	0.020	-.774**	.655**	0.374
AEHT	0.393	0.160	.585*	.723**	0.260	0.193	.477*	0.181	.780**	-.662**	-.491*
AP	.534*	0.145	.511*	.615**	0.248	0.045	0.339	0.177	.708**	-.657**	-0.368
AABP	0.165	-0.008	.525*	.591**	0.038	0.043	0.301	0.005	.755**	-.635**	-0.314
AAWS	-0.232	-.616**	-0.202	-0.146	-0.205	-0.397	-0.316	-.600**	0.186	-0.291	-0.141
AAT	.541*	0.071	.518*	.583*	0.217	0.213	0.416	0.108	.586*	-0.438	-.606**
AAVP	.583*	0.147	.530*	.614**	0.294	0.250	0.461	0.183	.595**	-0.468	-.584*
AARH	0.370	0.245	0.163	0.168	0.345	0.402	0.345	0.260	-0.073	0.147	-0.330
AALT	.526*	0.127	.561*	.599**	0.307	0.274	.469*	0.160	.535*	-0.400	-.664**
ACT≥10	.498*	-0.058	0.335	0.420	0.089	0.067	0.241	-0.017	.518*	-0.413	-0.458
ASD	-.526*	-.541*	-0.437	-0.399	-.550*	-.572*	-.575*	-.551*	-0.053	-0.048	0.428

Table S1 Correlations between the ecological factors and active ingredient contents of *S. miltiorrhiza* cover the eastern and western sites. AEHT-annual extreme high temperature, AP-annual precipitation, AABP-annual average barometric pressure, AAWS-annual average wind speed, AAT-annual average temperature, AAVP-annual average atmospheric vapor pressure, AARH-annual average atmospheric relative humidity, AALT-annual average lowest temperature, ACT≥10-annual cumulative temperature above 10 ℃, ASD-annual sunshine duration. RA-rosmarinic acid, SAB-salvianolic acid B, DTS-dihydrotanshinone, CTS-cryptotanshinone, TSI-tanshinone I, TSIIA-tanshinone IIA, TTS-total tanshinone, TPA-total phenolic acid. Pearson's two-tailed correlation analysis was performed using IBM SPSS Statistics 23 software. * Significant at $P < 0.05$, ** Significant at $P < 0.01$.

Eastern sites	RA	SAB	DTS	CTS	TS Ⅰ	TS ⅡA	TTS	TPA	CTS/TTS	TS ⅡA/TTS	TPA/TTS
Latitude	-.932**	-0.334	-0.472	-0.526	-0.516	-0.190	-0.395	-0.396	-.842**	.759*	0.536
Longitude	-0.044	0.193	-0.245	-0.127	-0.166	-0.207	-0.179	0.181	-0.100	0.005	0.576
Elevation	-0.251	-0.382	-0.564	-0.530	-0.575	-0.505	-0.555	-0.386	-0.266	0.127	.715*

AEHT	.695*	0.337	0.477	0.593	0.631	0.309	0.501	0.379	.724*	-0.652	-.721*
AP	.868**	0.475	0.412	0.513	0.496	0.139	0.360	0.524	.808**	-.755*	-0.290
AABP	-0.117	0.290	0.115	0.033	0.115	0.112	0.083	0.267	-0.300	0.255	0.026
AAWS	-0.167	-.726*	-0.430	-0.479	-0.272	-0.498	-0.487	-.707*	-0.054	-0.185	0.070
AAT	.850**	0.323	0.489	0.574	0.537	0.251	0.450	0.379	.831**	-.717*	-0.648
AAVP	.947**	0.426	0.464	0.569	0.508	0.213	0.426	0.484	.858**	-.756*	-0.481
AARH	0.556	0.427	0.196	0.290	0.236	0.078	0.197	0.453	0.425	-0.397	0.075
AALT	.855**	0.408	0.538	0.635	0.590	0.307	0.512	0.460	.841**	-.710*	-.670*
ACT≥10	.809**	0.136	0.300	0.396	0.347	0.053	0.251	0.196	.818**	-.757*	-0.465
ASD	-.829**	-.746*	-0.577	-0.659	-0.564	-0.349	-0.533	-.780*	-.702*	0.551	0.311

Table S2 Correlations between the ecological factors and active ingredient contents of *S. miltiorrhiza* in the eastern sites AEHT-annual extreme high temperature, AP-annual precipitation, AABP-annual average barometric pressure, AAWS-annual average wind speed, AAT-annual average temperature, AAVP-annual average atmospheric vapor pressure, AARH-annual average atmospheric relative humidity, AALT-annual average lowest temperature, ACT≥10-annual cumulative temperature above 10 ℃, ASD-annual sunshine duration. RA-rosmarinic acid, SAB-salvianolic acid B, DTS-dihydrotanshinone, CTS-cryptotanshinone, TSI-tanshinone I, TSIIA-tanshinone IIA, TTS-total tanshinone, TPA-total phenolic acid. Pearson's two-tailed correlation analysis was performed using IBM SPSS Statistics 23 software. * Significant at $P < 0.05$, ** Significant at $P < 0.01$.

Western sites	RA	SAB	DTS	CTS	TS Ⅰ	TSⅡA	TTS	TPA	CTS/TTS	TSⅡA/TTS	TPA/TTS
Latitude	0.049	0.417	0.058	0.095	0.297	0.075	0.118	0.393	-0.035	-0.317	0.373
Longitude	0.222	0.477	0.426	.675*	.812**	0.602	.681*	0.462	0.134	-0.155	-.684*
Elevation	-0.519	-0.645	-0.662	-.881**	-.719*	-.897**	-.903**	-0.642	-0.167	-0.381	.689*
AEHT	0.558	.706*	0.570	.881**	0.590	.821**	.837**	.702*	0.342	0.282	-0.425
AP	0.394	0.233	0.501	0.489	0.192	0.527	0.488	0.248	0.083	0.588	-0.601
AABP	0.619	.758*	.780*	.935**	.755*	.965**	.965**	.754*	0.124	0.473	-.669*
AAWS	-0.261	-0.468	-0.391	-0.414	-0.027	-0.197	-0.241	-0.457	-.710*	0.289	-0.273
AAT	0.419	0.140	0.403	0.455	0.108	0.489	0.441	0.164	0.145	0.598	-0.563

AAVP	0.437	0.230	0.462	0.576	0.339	0.620	0.591	0.249	0.080	0.561	-.732*
AARH	0.299	0.111	0.357	0.486	0.410	0.544	0.529	0.126	-0.004	0.407	-.771*
AALT	0.342	0.128	0.484	0.460	0.191	0.507	0.467	0.146	0.080	0.562	-0.663
ACT≥10	0.339	-0.007	0.169	0.204	-0.020	0.328	0.253	0.020	-0.140	.712*	-0.414
ASD	-0.370	-0.399	-0.638	-.730*	-0.550	-.696*	-.713*	-0.401	-0.293	-0.206	.692*

Table S3 Correlations between the ecological factors and active ingredient contents of *S. miltiorrhiza* in the western sites. AEHT-annual extreme high temperature, AP-annual precipitation, AABP-annual average barometric pressure, AAWS-annual average wind speed, AAT-annual average temperature, AAVP-annual average atmospheric vapor pressure, AARH-annual average atmospheric relative humidity, AALT-annual average lowest temperature, ACT≥10-annual cumulative temperature above 10 ℃, ASD-annual sunshine duration. RA-rosmarinic acid, SAB-salvianolic acid B, DTS-dihydrotanshinone, CTS-cryptotanshinone, TSI-tanshinone I, TSIIA-tanshinone IIA, TTS-total tanshinone, TPA-total phenolic acid. Pearson's two-tailed correlation analysis was performed using IBM SPSS Statistics 23 software. * Significant at $P < 0.05$, ** Significant at $P < 0.01$.
